# Reviewed article: Ribeiro LF; Moura ED; Guimarães PC; Rosa TA. Completeness and clinical-epidemiological profile of notifications of venomous animal envenomations: a descriptive analysis in the Centro-Sul region of the State of Rio de Janeiro, 2020-2023. Epidemiol Serv Saude. 2026:35;e20250541

**DOI:** 10.1590/S2237-96222026v35e20250541.a

**Published:** 2026-11-09

**Authors:** João Paulo Cola

**Affiliations:** 1 Universidade Federal do Espírito Santo, Vitória, ES, Brazil Universidade Federal do Espírito Santo Vitória ES Brazil

## Peer review A

### Questionnaire

Does the manuscript contain new and significant information to justify publication? Yes 

Does the Abstract (Summary) clearly and accurately describe the content of the article? No

Is the problem significant and concisely stated? No

Are the methods described comprehensively? No

Are the interpretations and conclusions justified by the results? No

Is adequate reference made to other work in the field? No

### Manuscript Structure:

Length of article is: Adequate

Number of tables is: Adequate

Number of figures is: Adequate

### Rating

Interest Average

Quality Poor

Originality Below Average

Overall Poor

Recommendation: **Reject**

## Comments

Comentários críticos:

1. A pergunta de pesquisa encontra-se bem definida e estruturada conforme a estratégia PICO.
2. O delineamento do estudo poderia ser revisto a fim de adequá-lo de forma mais precisa à questão proposta. Sugere-se avaliar se o trabalho se enquadra em um estudo transversal e se contempla, ainda, uma possível abordagem qualitativa, conforme descrito no manuscrito.
3. Observa-se uma carência de detalhamento metodológico. Recomenda-se descrever os métodos de forma mais completa, incluindo o contexto da pesquisa (considerando que parte dele já se encontra na introdução e deve ser alocado nos métodos), os participantes, o detalhamento das variáveis analisadas, a fonte dos dados e a descrição pormenorizada da análise realizada (quais medidas foram calculadas e de que forma). Esse aprimoramento permitirá maior clareza e possibilitará que outros pesquisadores repliquem o estudo.
4. O método utilizado para a análise de completude pode ser mais bem adequado. Recomenda-se adotar métodos padronizados para avaliar e classificar a completude dos dados. Entre as referências mais utilizadas, destacam-se as recomendações do CDC, no Updated guidelines for evaluating public health surveillance systems: recommendations from the guidelines working group, e da CEPAL, no documento Gestión orientada a asegurar la calidad de los datos en los institutos nacionales de Estadística. A adoção desses referenciais pode contribuir para maior clareza na interpretação dos resultados e para a comparabilidade das análises. Os autores podem, ainda, utilizar como referência outros estudos publicados na RESS que analisaram a completude, o que contribuirá para alinhar a análise às práticas adotadas em pesquisas anteriores.
5. As tabelas e figuras estão bem elaboradas, de fácil compreensão e complementam o texto de forma eficaz. Contudo, a Tabela 1 requer ajustes, como a indicação do número total (N) de cada tipo de animal envolvido nos acidentes e a inclusão de uma coluna com a totalização das fichas analisadas. Na Figura 1, recomenda-se verificar a formatação decimal do eixo X, pois provavelmente ocorreu a substituição da vírgula pelo ponto. Já nos títulos das Tabelas 2 e 3, é necessário revisar o N informado, uma vez que não corresponde aos dados apresentados. Na tabela 3 revisar o padrão de apresentação da média com desvio padrão.
6. A conclusão deve ser consistente e sustentada pelos resultados apresentados e responde de maneira adequada à pergunta de pesquisa. Não está descrita no texto ao final de discussão para o fechamento do estudo.
7. Revisar o resumo para apresentar o estudo de forma clara e lógica. Na seção de métodos, é importante explicitar as fontes de informação utilizadas, bem como indicar quais medidas de frequência foram calculadas e de que maneira, de modo a apoiar a interpretação dos resultados. Os principais achados devem ser apresentados de forma concisa e numérica, em consonância com os métodos descritos e com o objetivo do estudo. Por fim, a conclusão deve responder diretamente ao objetivo proposto, fundamentando-se nos resultados apresentados. Esse cuidado é fundamental porque o resumo é, muitas vezes, a seção mais consultada do manuscrito e determinante para a compreensão rápida da relevância do estudo.
8. Verificar a pertinência de modificar o DeCS de “Sistemas de Informação” para “Sistemas de Informação em Saúde”, descritor mais adequado por estar em consonância com a indexação utilizada.

Comentários importantes:

1. Destacar de forma mais clara a originalidade do estudo, apresentando o estado da arte sobre a temática até o momento e evidenciando o que a pesquisa pretende trazer de novo, explicitando a lacuna que busca preencher. Como sugestão, o aprofundamento da análise da completude das fichas e da avaliação do uso de soros adequados, considerando a classificação dos casos, reforçaria a contribuição do trabalho para o avanço do conhecimento na área.
2. Aprofundar a discussão do potencial dos achados para aplicação em diferentes contextos, indo além do interesse local. É importante considerar em que medida os resultados podem ser generalizados para outros cenários e populações. Em especial, recomenda-se que os autores expliquem se os achados relacionados à sazonalidade dos acidentes podem ser extrapolados para outras populações ou se refletem características específicas do contexto estudado.
3. Por se tratar de um estudo descritivo, é fundamental justificar a relevância dos achados, explicitando como os resultados contribuem para responder às lacunas existentes no conhecimento sobre a temática.
4. Estudos que utilizam dados secundários são suscetíveis a vieses de informação e erros de preenchimento, o que pode comprometer a validade dos resultados. Recomenda-se que os autores discutam essas limitações de forma explícita, detalhando as estratégias empregadas para minimizar os vieses e explicar como a validade foi assegurada.
5. Observa-se uma lacuna no detalhamento dos dados incluídos na análise, especialmente quanto às variáveis utilizadas e ao processo de alimentação do banco de dados. O manuscrito deve apresentar uma descrição clara do processo de vigilância dos acidentes por animais peçonhentos, incluindo quando a notificação deve ser realizada, quem é o responsável por notificá-la, como ocorre a qualificação das notificações, se há conferência dos dados em prontuários e se existe orientação da equipe de plantão ou de serviços de referência para o uso de soros antivenenos. Esse esclarecimento é essencial para compreender potenciais vieses e assegurar a validade das análises.
6. Importante ampliar a discussão sobre as implicações práticas dos achados relacionados à assistência. Destaca-se, em especial, o uso desnecessário de ampolas de soro antiveneno, que merece maior aprofundamento quanto às suas consequências na prática assistencial e no processo de cuidado. Recomenda-se explorar medidas e ferramentas para modificar esse cenário, considerando protocolos, capacitação das equipes e estratégias de uso racional de insumos. Esse aspecto constitui um diferencial e um dos potenciais do estudo, sendo relevante valorizá-lo na discussão, de modo a evidenciar as contribuições concretas para a qualificação da assistência e para a gestão em saúde.
7. A discussão sobre a incompletude dos dados pode ser fortalecida ao incluir reflexões sobre seus impactos tanto na assistência quanto na gestão em saúde. A ausência ou falha no registro compromete a tomada de decisão, a definição de condutas terapêuticas adequadas e o planejamento de ações de vigilância. Recomenda-se, ainda, apontar soluções factíveis para reduzir a incompletude, como a adoção de sistemas informatizados mais intuitivos e mecanismos de monitoramento da qualidade dos registros. Esse aprofundamento contribuirá para realçar a relevância prática do estudo e oferecer subsídios para o aprimoramento dos processos de informação em saúde.
8. Revisar o título para ser mais adequado ao objetivo de estudo. Sugestão de utilizar “Completude e perfil clínico e epidemiológico das notificações de acidentes por animais peçonhentos em uma cidade polo para distribuição de soros antivenenos: um estudo transversal.

Comentários desejáveis:

1. Revisar a terminologia e as denominações das variáveis utilizadas no estudo, assegurando que estejam em conformidade com os padrões científicos e sejam compreensíveis ao público leitor. Em especial, termos técnicos relacionados ao tipo de animal envolvido - como loxoscelismo, phoneutrismo, entre outros - devem ser devidamente conceituados na metodologia ou em momento oportuno ao longo do texto, favorecendo a compreensão dos achados. Detalhar de forma mais clara as variáveis utilizadas na seção de metodologia contribuirá para maior precisão conceitual e melhor interpretação dos resultados apresentados.
2. Revisar o texto para assegurar maior clareza, precisão e objetividade na linguagem. Recomenda-se evitar expressões imprecisas, como “aproximadamente 40%” ou “quase 300 acidentes”, substituindo-as por valores absolutos e percentuais exatos. Essa prática confere maior rigor científico e consistência à apresentação dos achados.
3. Verificar se todas as citações de fato apoiam as afirmações realizadas. Observa-se que, em alguns trechos, especialmente aqueles relacionados ao sistema de informação e à ficha de notificação, foram utilizadas referências genéricas (2, 3, 4, 5, 8, 11), que não parecem subsidiar de forma adequada as afirmações apresentadas. Sugere-se revisar e atualizar as citações, priorizando fontes específicas e pertinentes que fortaleçam a fundamentação teórica do estudo.
4. Revisar a lista de referências, assegurando que ela contemple os trabalhos mais relevantes e recentes sobre a temática. Observa-se que a maioria dos estudos citados foi publicada há mais de cinco anos. Os autores poderiam se empenhar em realizar uma revisão de literatura mais robusta, incorporando achados atuais que subsidiem de forma mais consistente a discussão.

